# Editorial: Uveitis: Insights into pathogenesis and treatment

**DOI:** 10.3389/fmed.2022.1033817

**Published:** 2022-11-24

**Authors:** Alessandra Soriano, Marco Soriano, Rocco Oliva, Wendy M. Smith

**Affiliations:** ^1^Gastroenterology Division and IBD Center, Department of Internal Medicine, Azienda Unità Sanitaria Locale—IRCCS, Reggio Emilia, Italy; ^2^School of Medicine, “Luigi Vanvitelli” University, Naples, Italy; ^3^School of Medicine, Modena and Reggio Emilia University, Modena, Italy; ^4^Department of Ophthalmology, Mayo Clinic, Rochester, MN, United States

**Keywords:** uveitis, epidemiology, pathogenesis, treatment, autoimmunity

Uveitis refers to a heterogeneous group of intraocular inflammatory diseases of the uvea and adjacent structures, and is one of the leading causes of visual impairment and preventable blindness. Genetic predisposition, cytokines signatures, T and B cell involvement and environmental factors seem to be involved in its pathogenesis.

Diagnosis of uveitis can be challenging. A multidisciplinary approach is usually needed to improve identification and fast-track management, especially in the case of non-infectious forms of uveitis. The diagnosis of uveitis can be challenging. Primary ocular conditions must be differentiated from systemic inflammatory diseases, infectious uveitis, and masquerades of inflammation such as malignancy. A multidisciplinary approach to diagnosis and management can improve the prognosis for control of inflammation and preservation of vision.

With the Research Topic on Frontiers in Medicine titled “*Uveitis: Insights into pathogenesis and treatment*,” we gathered experts in the field of ocular immunology to identify the directions of clinical and translational research in non-infectious uveitis. Insights on the epidemiology, pathogenesis, and treatment of noninfectious uveitis may contribute to further understanding of other inflammatory conditions such as inflammatory bowel disease, Behcet's disease, and sarcoidosis. Finally, this Research Topic offers the opportunity to identify additional questions to answer in the field of uveitis.

In their systematic review performed in a 50-year period (1951–2021), Joltikov and Lobo-Chan found very few epidemiologic studies specifically focused on non-infectious uveitis. Data on incidence and prevalence are still “provisional,” as they considerably vary worldwide due to the fact that the few available studies include also infectious and idiopathic forms. What became clear is that sexual dimorphism exists even in non-infectious uveitis, as observed in most of the immune-mediated systemic conditions they are frequently related to. A number of factors need to be considered including ethnicity, age, smoking, hormone levels, hormone replacement therapy, and eventually gut microbiome, which represents another new frontier to be explored as potential modifier of the gut and systemic immune environment (1).

The importance of understanding the cellular composition and functionality of the uveal immune environment has been underlined by Reekie et al. in their comprehensive work on what is the current knowledge on the roles played by different cell types to contribute to immune homeostasis and tolerance in the uvea. Both leukocyte and stromal biology in this peculiar microanatomical niche need to be further investigated, as it remains unclear how these two populations interact to permit the inflammatory “burden,” where required.

The role of fibroblasts in the uvea remains unclear, but it seems to differ from their roles in other microenvironments such as the gut, where transcriptome analysis has shown that the fibroblast populations vary along the crypt-villus axis. Similarly, in IBD single-cell profiling over colonic mesenchymal cells revealed four subsets of fibroblasts expressing divergent transcriptional regulators and functional pathways.

Nowadays we know that niche population located in proximity to epithelial crypts fuels inflammation and barrier dysfunction (2). We also know that uveitis (together with episcleritis and scleritis) can be an extra-intestinal manifestation of IBD which may precede or follow systemic disease onset. Interestingly enough, in this particular case, we know that usually by treating the underlying IBD, ocular symptoms tend to improve accordingly. However, we still do not know why in other cases ocular manifestations (i.e., uveitis or scleritis) have a clinical course independently from the systemic disease course of IBD.

As Reekie et al. extensively discussed, some open questions could be answered by integrating evidence from studies already performed on other systemic immune-mediated inflammatory diseases, and data coming from advanced investigative techniques such as single-cell transcriptomics and high-multiplexed imaging.

Additional new perspectives on the immunopathogenesis of another multisystem T-lymphocyte-mediated granulomatous autoimmune disease, Vogt-Koyanagi-Harada (VKH) disease, have been provided by El-Asrar et al. Analysis of the aqueous humor samples from VKH patients with uveitis may help identify potential prevalent cell subsets and novel biomarkers (3). The authors were able to demonstrate in previous original research significantly high levels of CXCL13 (a B cell attracting chemokine 1), BAFF, APRIL, and TWEAK (i.e., cytokines promoting B cell survival differentiation and proliferation) concentrations in the aqueous humor samples of patients with VKH disease (4, 5). By using a “bench-to-bedside” approach, they suggested the use of rituximab, a chimeric mouse/human monoclonal antibody against the pan-B cell marker CD20 in refractory uveitis associated with VKH disease.

Finally, the efficacy and safety of adalimumab, a fully human anti-TNF alpha monoclonal antibody, in treating pediatric non-infectious chronic anterior uveitis patients with peripheral retinal vascular leakage, has been investigated in a pilot study by Song et al. The authors show promising results in controlling intraocular inflammation and peripheral vascular leakage in pediatric patients. Of note, among the 20 enrolled Chinese pediatric patients with anterior uveitis, 17 cases were defined as idiopathic, two were associated with juvenile idiopathic arthritis, and one with Blau syndrome.

In conclusion, this Research Topic certainly provides us with abundant “food for thought” on how much research there is still to be done in the field of ocular immunology, both in adult and in pediatric populations.

Over the years it has become clear that aspiring for real precision medicine means integrating knowledge from various disciplines. Non-infectious uveitis represents one of the most paradigmatic examples of how close collaboration between the various specialists of different branches of medicine shed light on what still remains obscure.

## Author contributions

All authors listed have made a substantial, direct, and intellectual contribution to the work and approved it for publication.

## Conflict of interest

The authors declare that the research was conducted in the absence of any commercial or financial relationships that could be construed as a potential conflict of interest.

## Publisher's note

All claims expressed in this article are solely those of the authors and do not necessarily represent those of their affiliated organizations, or those of the publisher, the editors and the reviewers. Any product that may be evaluated in this article, or claim that may be made by its manufacturer, is not guaranteed or endorsed by the publisher.
